# Development and validation of a questionnaire to assess the knowledge of mechanical ventilation in urgent care among students in their last-year medical course in Brazil

**DOI:** 10.6061/clinics/2019/e663

**Published:** 2019-10-14

**Authors:** Fernando Sabia Tallo, Simone de Campos Vieira Abib, Andre Luciano Baitello, Renato Delascio Lopes

**Affiliations:** I Departamento de Cirurgia, Universidade Federal de Sao Paulo (UNIFESP), Sao Paulo, SP, BR; II Faculdade de Medicina de Sao Jose do Rio Preto (FAMERP), Sao Jose do Rio Preto, SP, BR; III Duke Clinical Research Institute, Duke University Medical Center, Durham, North Carolina, US

**Keywords:** Mechanical Ventilation, Medical Education, Psychometrics, Emergencies

## Abstract

**OBJECTIVE:**

To develop and validate a questionnaire to assess the knowledge of mechanical ventilation among final-year medical students in Brazil.

**METHODS:**

A cross-sectional study conducted between October 2015 and October 2017 involving 554 medical students was carried out to develop a questionnaire for assessing knowledge on mechanical ventilation. Reproducibility was evaluated with the intraclass correlation coefficient, internal consistency was evaluated with Cronbach’s alpha, and construct validation was evaluated with a tetrachoric exploratory factor analysis. To compare the means of the competences among the same type of assessment tool, the nonparametric Friedman test was used, and the identification of the differences was obtained with Dunn-Bonferroni tests.

**RESULTS:**

The final version of the questionnaire contained 19 questions. The instrument presented a clarity index of 8.94±0.83. The value of the intraclass correlation coefficient was 0.929, and Cronbach’s alpha was 0.831. The factor analysis revealed five factors associated with knowledge areas regarding mechanical ventilation. The final score among participants was 24.05%.

**CONCLUSION:**

The instrument has a satisfactory clarity index and adequate psychometric properties and can be used to assess the knowledge of mechanical ventilation among final-year medical students in Brazil.

## INTRODUCTION

The increased time patients spend on mechanical ventilation in the emergency department and the inadequate approach to mechanical ventilation increase mortality and length of hospital stay (1). In addition, many patients require prolonged acute mechanical ventilation (>96 hours) in the emergency department, and physicians from multiple specialties take care of these patients (2). Many of these physicians feel uncomfortable when manipulating these patients and often transfer this responsibility to other professionals (3).

In Brazil, many newly graduated physicians work in the emergency room with severe mechanical ventilation patients but have little training in this field (4). There is no existing literature that has evaluated their knowledge of mechanical ventilation.

The objective of this study was to develop and validate an instrument to evaluate the knowledge of mechanical ventilation in the emergency room by a final-year medical students in Brazil.

## METHODS

Following approval by the Research Ethics Committee of the Universidade Federal de São Paulo (UNIFESP) (report number CAEE 17690513.7.0000.5505), a descriptive cross-sectional study was initiated in October 2015 and completed in October 2017. A literature review was conducted in the PUBMED database with the following MeSH terms: artificial, ventilation, emergency department, medical education, and psychometrics. The search resulted in 1253 articles, and 66 were selected, with 15 being included in the final study. A panel of twenty mechanical ventilation “specialists” (expert group) was formed to develop a questionnaire to assess the knowledge of mechanical ventilation among sixth-year students of the Brazilian medical course (APPENDIX). Medical specialists who published books, chapters or articles on mechanical ventilation or who had teaching experience in intensive care, pulmonology or anesthesiology were considered. The second sample included 60 students in their last-year medical course at UNIFESP and 60 intensive care unit (ICU) physicians with specialist degrees (validation cohort group). The third sample involved 554 medical students in their sixth-year undergraduate course (medical students group). All of the questionnaire respondents signed the voluntary informed consent form before participation in the study.

The validation methods followed the Consensus-based Standards for the Selection of Health Status Measurement Instruments (COSMIN) standard checklist (5).

Regarding content validation, the educational objectives were developed through the Delphi technique (6). The following subjects were selected: respiratory physiology, beginning and maintenance of mechanical ventilation, modes and modalities of mechanical ventilation, and complications and monitoring.

To evaluate the clarity and relevance of each item of the instrument, an interval score of 1 to 10 was created for the two components. For clarity, a score between 1 and 4 was considered confusing; a score between 5 and 7 was considered not very clear; and a score between 8 and 10 was considered clear. For relevance, a score between 1 and 4 was considered irrelevant; a score between 5 and 7 was considered not very relevant; and a score between 8 and 10 was considered relevant. The items were considered for the final version only if they achieved a core of 8-10 points.

Below each component, there was a space for suggestions regarding the content and semantics of the proposed items. The indexes of clarity and relevance were obtained through the mathematical averages of the sums of the score given by the professionals. The statements with relatively low clarity and/or relevance (index below 8.0) were replaced or reworded.

The second version of the questionnaire was evaluated for clarity by twenty medical students with a method similar to that used by the health professionals. The items with scores equal to or less than 8.0 were reformulated, and the final version of the questionnaire, which underwent a process of construct validation and reproducibility, was generated. The established scores for the questions were as follows: correct=1; incorrect or do not know=0. The sum of the question scores represented the final score (7).

The participants in the expert group, sixty medical students and sixty physicians board-certified in intensive medicine, answered the final version of the questionnaire. The final scores of the two groups were compared using the Mann-Whitney test.

Reproducibility was evaluated with 60 (expert group) medical students who agreed to answer the questionnaire twice, with an interval of 14 days (test and retest). The statistical analysis was performed using the interclass correlation coefficient (8). The students did not receive specific information on mechanical ventilation during this interval or any information regarding their performance. For the interclass correlation coefficient analysis, the total scores generated by the instrument were used based on a value higher than 0.8.

The third sample (medical students group) comprised students from 10 educational institutions in Brazil: UNIFESP, Federal University of Bahia, Federal University of Mato Grosso do Sul, Federal University of Goiás, Federal University of Rorâima, UniEvangélica Medical School, Medical School of São José de Rio Preto, Medical School of Votuporanga, Universidade Nove de Julho (UNINOVE) Medical School, Universidade de Salvador (UNIFACS), Roberto Santos Hospital, and students and residents who attended the Brazilian Society of Internal Medicine (SBCM) course on mechanical ventilation from several educational institutions. This sample evaluated the construct validity and the internal consistency of the instrument. All the students completed the questionnaire in person within 30 minutes. The instrument contained 20 multiple-choice questions and five self-reporting questions about mechanical ventilation teaching and practice during the undergraduate course. Correlation analyses with the categorical variables were performed.

### Construct Validation

To validate the construct, an exploratory factor analysis was performed based on the tetrachoric correlation matrix to evaluate the dimensionality suggested by the items of the dichotomic questionnaire. The exploratory factor analysis was performed with the main component method and VARIMAX orthogonal rotation (9,10).

The overall and the subdimension internal consistency was analyzed via Cronbach’s alpha coefficient (11).

The sum of the scores of the correct answers in the respective dimensions were generated and were rescaled in such a way that they varied from 0 (minimum) to 100 (maximum).

Once mean differences were detected, the differences were identified with Dunn-Bonferroni tests, with the level of global significance maintained.

The categorical items described were as follows: program schedule with or without mechanical ventilation course; hours spent searching for mechanical ventilation information; number of patients on mechanical ventilation assisted per week; level of comfort in the handling of patients on mechanical ventilation; and professional who handles patients on mechanical ventilation in the emergency room.

## RESULTS

The expert group consisted of 18 specialists; after three rounds of item evaluation, the questionnaire reached clarity and relevance indexes ranging from 8-10. The final questionnaire was answered by the validation cohort comprising 60 medical students from UNIFESP and 60 attending physicians. The final scores among the members of the expert group were compared using the Mann-Whitney test, shown in Table 1.

Subsequently, the questionnaire was answered by 554 medical students from eleven medical schools (Table 2).

The number of questionnaires administered was 592; 554 were included. Only those with at least 90% completion were considered.

Reproducibility was tested with the interclass correlation coefficient and had a value of 0.929.

**Figure 1:**
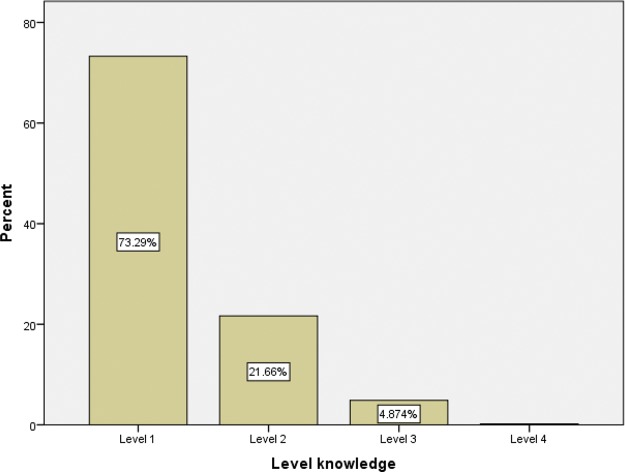
Students’ knowledge of mechanical ventilation. Level 1: ≤25% of the score, Level 2: >25% but ≤50% of the score, Level 3: >50% but ≤5% of the score, Level 4: >75% to 100% the score.

Among the respondents, 15.2% obtained the minimum score of “zero”, and none reached the maximum score of “twenty”. The loss percentage was 2.2% among all the questionnaires considered. Of the sample, 83% of the students did not have any mechanical ventilation course on their medical school schedule. The level of knowledge based on the percentages of the final scores is presented in Figure 1. The scores obtained by the students, stratified by different factors, are shown in Table 3.

Moderate positive correlations were found between the level of knowledge and the following variables: hours of mechanical ventilation in the undergraduate course (rho=0.552, *p*<0.001) and information on mechanical ventilationfrom other sources (rho=0,506, *p*<0.001). The professionals who initiated mechanical ventilation in the emergency department were physiotherapists according to 63% of the respondents, and 82.5% of the respondents never participated in the care of a patient on mechanical ventilation.

The exploratory factor analysis is shown in Table 3 and contains the following factors: acute respiratory distress syndrome (ARDS) factor, chronic obstructive pulmonary disease (COPD) factor, complications factor, modality factor, and respiratory factor. We observed the existence of five factors that together accounted for 86.5% of the total explained variance among the items; the eigenvalues of which were higher than 1.0. Item 11 was excluded due to low commonality.

The first factor, the “ARDS factor”, covered five items involving the following areas of knowledge: the concept of plateau pressure, the concept of respiratory system compliance, positive end-expiratory pressure (PEEP), and mechanical ventilation strategies in ARDS patients. The items explained 24.9% of the total variance.

Factor two, the “COPD factor”, covered four items: the concept of auto-PEEP, auto-PEEP complications, behavior of patient on auto-PEEP in the emergency room, principles of ventilation in COPD patients, and indications for noninvasive ventilation. The third factor, the “Complications factor” covered five items: complications of mechanical ventilation, concept of airway resistance, arterial blood gas testing, and auto-PEEP measurements. The fourth factor, the “modality factor”, covered three items involving concepts of the most commonly used modalities. The last factor, the “respiratory factor”, involved concepts of respiratory physiology. After obtaining the different knowledge factors in the tetrachoric factor analysis, the means of each factor were compared with the nonparametric Friedman test due to the violation of the assumption of normality. In the distribution of scores, the highest performance was on “factor 5” and the lowest was on “factor 1” (Table 4).

The internal consistency of the instrument was measured by Cronbach’s alpha and had a value of 0.831.

## DISCUSSION

Previous studies have developed instruments for assessing the knowledge of mechanical ventilation among emergency and clinical residents and nurses (12-15). Our study is the first to validate an instrument to assess the knowledge of mechanical ventilation among medical students.

The data on the internal consistency and reproducibility demonstrated the homogeneity and stability of the instrument and the possibility of obtaining similar and accurate results (16).

The tetrachoric exploratory factor analysis revealed five factors, a finding that demonstrates the multidimensional nature of knowledge on mechanical ventilation. The clusters were related to specific aspects, such as mechanical ventilation in ARDS, COPD, complications of mechanical ventilation, physiology, and the beginning and maintenance of mechanical ventilation.

A very low final score average among the students was observed. This level of knowledge was associated with the absence of self-reported mechanical ventilation teaching programs. The instrument showed that the scores increased as the number of hours of mechanical ventilation activities performed by students increased.

The factor related to physiology obtained the best score. However, the factors that were related to specific knowledge of mechanical ventilation obtained very low scores. The poor performance in factor three, which was related to complications in patients on mechanical ventilation, is highlighted.

Our study has some limitations; the sample of students was not probabilistic, although the study involved a large number of students. Moreover, the relations of the scores measured by the instrument and the time spent learning about mechanical ventilation were based on self-reported, nonmeasured information.

In conclusion, the questionnaire called “Questionnaire on the knowledge of mechanical ventilation in the emergency room by sixth-year medical school students in Brazil” presented the psychometric properties necessary to serve as an evaluation tool for this population. 

## APPENDIX

### ASSESSMENT OF KNOWLEDGE OF MECHANICAL VENTILATION IN URGENT CARE AND EMERGENCY DEPARTMENTS

Instructions to answer the questionnaire:

1. There is only one correct answer.
2. If you are not sure about the answer or are undecided between two or more choices, select “I don’t know”.
3. The following abbreviations are used:

PEEP - Positive end-expiratory pressure, COPD - Chronic obstructive pulmonary disease, TV - Tidal volume, RR - Respiratory rate, VCV - Volume-controlled ventilation, A-C -Assist-control mode, FiO2 - Fraction of inspired oxygen, Pplat - Plateau pressure, PIP - Peak inspiratory pressure, IP- Inspiratory Pressure, GCS- Glasgow coma scale, bpm - breaths per minute, PaCO2- Partial pressure of carbon dioxide, V/Q- Pulmonary ventilation/perfusion ratio, ER- emergency room, ABG - Arterial blood gas.

NAME: _________________________________________________________ STATE: ______________________________________

ID: _______________________________________________ EMAIL: ____________________________________________________

**STUDENT ( ) RESIDENT 1^st^ YEAR ( ) RESIDENT 2^nd^ YEAR ( ) EMERGENCY PHYSICIAN ( ) SPECIALIST ( )**

1. Did you attend theoretical or practical classes on mechanical ventilation during your undergraduate education or medical residency?
  A. YES
  B. NO
2. How many hours did you spend obtaining information on mechanical ventilation (reading of articles, discussion in journal clubs, conferences/lectures, congresses, classes, etc.) during your undergraduate education or medical residency?
  A. 0-1
  B. 2-3
  C. 4-5
  D. More than 5
  E. I don’t know (Please provide your best estimate if possible)
3. How many patients on mechanical ventilation in the emergency room did you help treat during your medical course or during your medical residency? (participated in treatment, discussed or followed changes in ventilation parameters, or changed the parameters yourself with supervision)?
  A. Never
  B. Rarely (1-3 patients/month)
  C. Many times (4-9 patients/month)
  D. Frequently (>10 patients/month)
  E. I don’t know
4. How frequently do you feel comfortable managing mechanical ventilation patients, with questions about managing ventilated patients in the emergency room?
  A. Never
  B. Rarely
  C. Many times
  D. Always
  E. I don’t know
5. Who initiates and introduces changes in the mechanical ventilation of intubated patients in the emergency department where you work?
  A. Physiotherapist
  B. Nurse
  C. Resident who is training in the emergency department
  D. Physician in charge of the emergency department
  E. Physician who is not related to the emergency department (ICU staff, pulmonologist, etc.)
  F. I don’t know
6. Which statement is true about the Pressure Safety Valve (PSV)?
7.
  A. The patient has his/her respiratory rate set by the operator
  B. The patient adjusts his/her PEEP at each mechanical cycle
  C. The patient adjusts the volume received through their own effort
  D. The patient has constant flow at inhalation
  E. I don’t know
8. A female patient at the emergency department undergoes endotracheal intubation due to respiratory failure. Her ventilation (PCV) is adjusted with a PEEP of 10 cmH2O, a rate of 18, and FiO2 of 80%. Her ABG results showed a pH of 7.36; PaCO2 of 40; and PaO2 of 220. The following change should be made in the ventilator settings:
  A. The PEEP should be increased to 12
  B. The respiratory rate should be reduced to 16
  C. The pressure should be increased
  D. The FiO2 should be reduced to a value below 50%
  E. I don’t know
9. A patient with a decrease in consciousness due to an ischemic stroke is intubated at the emergency department. His predicted weight is 60 kg, his ventilation (VCV) is adjusted to 500 ml, with a PEEP of 5 cmH2O, RR of 25, and FiO2 of 40%. His ABG test shows a pH of 7.52; PaCO2 of 25; and PaO2 of 120. Among the statements below, which is the most ADEQUATE change that should be made in the ventilator parameters?
  A. Reduce the respiratory rate to approximately 20 bpm
  B. Increase the TV to approximately 600 ml
  C. Increase the FiO2 to approximately 80%
  D. Increase the PEEP to approximately 10 cmH2O
  E. I don’t know
10. A patient with a diagnostic suspicion of ARDS is started on mechanical ventilation. Which of the guiding principles for mechanical ventilation parameters should be used based on this diagnosis?
  A. Maintain FiO2 at 100% to maximize his oxygenation
  B. Reduce the PEEP to minimize alveolar barotrauma
  C. Maintain the Pplat <30 to minimize alveolar barotrauma
  D. Maintain a high TV to prevent respiratory acidosis
  E. I don’t know
11. The patient above has a predicted weight of 60 kg, his VCV parameters are as follows: volume of 525 ml, PEEP of 5 cmH2O, rate of 22, and FiO2 of 100%. His ABG tests show a pH of 7.35; PaCO2 of 41; and PaO2 of 95. His PIP is 38 cmH2O, and his plateau pressure is 34 cmH2O. His auto-PEEP is 0. The following change in the ventilation parameters should be made:
  A. Reduce his respiratory rate to reduce his PIP
  B. Reduce his TV to reduce his Pplat
  C. Reduce his PEEP because his oxygenation is adequate
  D. Increase his TV to help compensate for his metabolic acidosis
  E. I don’t know
12. Regarding the bedside monitoring of patients on noninvasive ventilation positive pressure (NIPPV) by healthcare professionals, choose the correct statement.
  A. A decrease in consciousness due to hypercapnia contraindicates the use of NIPPV
  B. The main monitoring parameter should be TV
  C. Eye irritation and claustrophobia are the most frequent complications
  D. The strategy of high flow prompts the rapid relief of respiratory distress
  E. I don’t know
13. A female patient with severe asthma is intubated due to respiratory failure at the ER. The ventilator (VCV) is adjusted to a volume 400 of ml, PEEP of 5 cmH2O, RR of 14, and FiO2 of 100%. Her flow-volume loop had the shape below on the ventilator screen. Which change in respiratory mechanics or physiology is this loop related to?
  **Figure 2:**
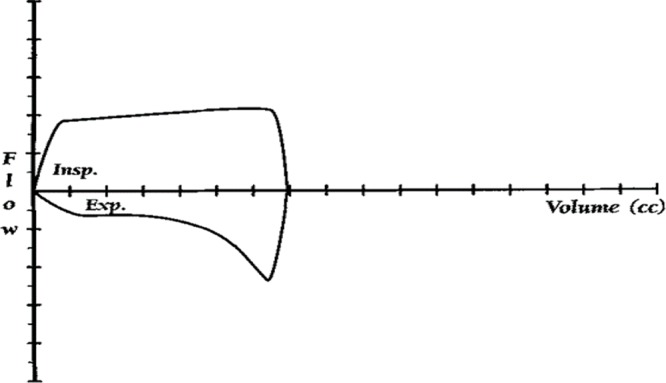
  A. Her respiratory system has high resistance
  B. Her respiratory system has low complacence
  C. Her respiratory system shows a large shunt
  D. Her respiratory system has a very low Pplat
  E. I don’t know
14. A severe COPD patient is intubated at the emergency department due to respiratory failure. After some minutes, the ventilator alarm for the maximum inspiratory pressure is triggered. What should be the physician’s immediate reaction?
  A. Perform a puncture to decompress the chest
  B. Remove the patient from the ventilator and ventilate him using a bag valve mask
  C. Immediately order a chest X-ray
  D. Administer a sedation bolus to the patient
  E. I don’t know
15. A 70-year-old woman with COPD arrives at the ER with a complaint of intense worsening of shortness of breath for three days. She reports “yellowish” sputum production in large quantities. On physical examination, she shows moderate respiratory distress using accessory muscles with GCS of 15. Her temperature is 100.4°F (axillary), her HR is 108, her RR is 30, and her BP is 115/75 mmHg. Vesicular murmurs are minor but bilaterally present with wheezing. The heart sounds are hypophonetic but regular, and there are no significant signs on the abdomen. A chest X-ray shows hyperinsuflation and a reduction in pulmonary vascularization in both lung fields. An ABG test performed with a nasal catheter of O2 at 3 L/min revealed the following: a pH of 7.29; PaCO2 of 62 mmHg, PaO2 of 50 mmHg, and SaO2 of 88%. In addition to monitoring, which of the following interventions would be MOST APPROPRIATE at this moment?
  A. Increased supplemental oxygen with beta-agonist therapy
  B. Continuous treatment with beta-agonists
  C. Endotracheal intubation and mechanical ventilation
  D. Noninvasive positive pressure ventilation (BIPAP)
  E. I don’t know
16. During treatment progression, physicians decide on orotracheal intubation. The patient is normotensive at 120/80 mmHg. Intubation was successful on the first attempt with an 8.0-mm tube. Soon after, the patient is ventilated with a frequency of 26 ventilations per minute with an inflating bag and unidirectional valve with an oxygen source at 15 L/min (AMBU). However, it is observed that the blood pressure drops to 70/40 mmHg. Among the statements below, which process is the MOST LIKELY cause of hypotension?
  A. Septic shock
  B. Pneumothorax
  C. Auto-PEEP
  D. Pulmonary thromboembolism
  E. I don’t know
17. The MOST APPROPRIATE intervention to improve cardiovascular impairment would be:
  A. Offer 500-1000 ml of IV crystalloids
  B. Decompression with jelco 14 in the left anterior intercostal space
  C. Maintain manual ventilation and allow that the patient to exhale
  D. Initiate dopamine at 10 mcg/kg/min
  E. I don’t know
18. The arterial pressure improved to 95/65 mmHg. Which ventilator parameters would be the most appropriate for this patient (60 kg predicted weight)?
  A. Assist-control volume with RR 20-22, TV 360 ml, PEEP 5 cmH2O, FiO2=100%
  B. Assist-control volume with RR 10-12, TV 480 ml, PEEP 5 cmH2O, FiO2=60%
  C. Assist-control volume with RR 20-22, TV 700 ml, PEEP 5 cm H2O, FiO2=60%
  D. Pressure assist-control ventilation with RR 15, IP 25 cmH2O, I/E R 1/1, PEEP 5 cm, H2O, FiO2100%
  E. I don’t know
19. Auto-PEEP measurement should be performed in patients prone to the dynamic hyperinflation phenomenon. How would you measure the “auto-PEEP” value in a patient with passive mechanical ventilation (sedated and paralyzed)?
  A. Measure the airway pressure during a pause of at least 2.0-3.0 seconds at the end of inhalation (and subtract the set of PEEPs)
  B. Measure the airway pressure during a pause of at least 2.0-3.0 seconds at final expiration (and subtract adjusted PEEP)
  C. Subtract the plateau pressure from the peak inspiratory pressure
  D. Multiply the caudal volume by the TV
  E. I don’t know
20. On assist/volume-controlled ventilation, if the respiratory rate is adjusted for 14 breaths per minute, and the TV at 500 mL, which would be the TV if the patient’s total respiratory rate was 20?
  A. 500 mL at each breath
  B. 500 mL during the 16 breaths defined and the rest determined by the patient’s effort
  C. The TV will be determined by the patient’s effort at each breath
  D. The TV will vary according to lung complacence
  E. I don’t know
21. A patient is on mechanical ventilation in the ER. You observe the graphs below on the ventilator screen. Choose the correct statement.
  **Figure 3:**
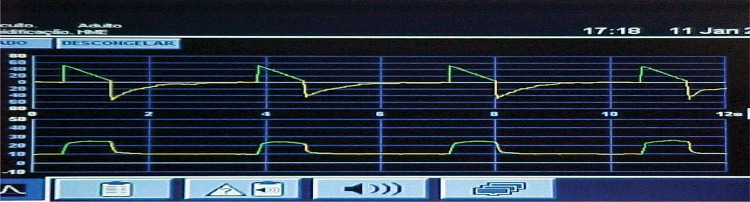
  A. The mode is PCV, controlled mode, the flow is ascending, TV and cycling are fixed
  B. The mode is VCV, assist/controlled mode, the flow is descending, TV is fixed and pressure-cycled
  C. The mode is PCV, assist/controlled mode, variable flow, TV is variable and time-cycled
  D. The mode is VCV, assist/controlled mode, variable flow, variable TV, pressure-cycled
  E. I don’t know
22. Consider a patient on mechanical ventilation in VCV mode with a squared flow wave. Regarding the airway peak pressure, plateau pressure and distension, choose the most APPROPRIATE statement.
  A. The peak pressure increases when the respiratory system compliance increases
  B. The plateau pressure increases when the airway resistance increases
  C. The distension pressure increases with increasing plateau pressure
  D. The PEEP increases when the airway resistance increases
  E. I don’t know
23. You are called to evaluate a patient on mechanical ventilation. The patient was in deep sedation; his SatO2 fell from 98 to 80% and his BP fell from 140/90 mmHg to 85/60 mmHg. The peak airway pressure increased from 38 cmH2O to 70 cmH2O, and his Pplat increased from 30 to 52 cmH2O. In the pulmonary auscultation, wheezing is present. There is a decrease in the left vesicular mucus. The sounds are hypophonetic and regular. Which of the following is the best-suited explanation for the changing airway pressures?
  A. Endotracheal tube occlusion
  B. Increase in bronchospasms
  C. Patient-ventilator asynchrony
  D. Hypertensive pneumothorax
  E. I don’t know
24. A 70-year-old woman is taken to the ER with dyspnea and a productive cough. She presents with a temperature of 102.0°F; RR of 34; HR of 120; BP of 80/50 mmHg; dry, sticky skin; and crackles in the left base. The institution protocol for sepsis has been initiated. She presents progressive worsening of the respiratory symptoms over the next 36 hours. A thorax radiogram reveals bilateral interstitial edema with no effusion. The patient uses a non-rebreather mask with an FIO2 of 90%-100%. Her blood gas show a pH of 7.28, PaCO2 of 35 mmHg, PaO2 of 55 mmHg, and SaO2 of 88%. Her predicted weight is 60 kg. which of the following ventilation paths is indicated by this patient?
  A. Ventilation with noninvasive positive pressure (BIPAP)
  B. Intubation and VCV with a RR of 20 and TV of 360
  C. Intubation and SIMV with a RR of 20 and TV of 700
  D. Intubation and VCV with a RR of 20 and TV of 700
  E. I don’t know
25. After 48 hours, the patient was ventilated with VCV/A-C. Her BP is 120/80 mmHg, and her HR is 92. The patient is in deep sedation (RASS -5), with a PIP of 40 cmH2O, Pplat of 24 cmH2O, FiO2 of 0.70 and PEEP of 5.0. Her ABG results show a pH of 7.28, PaCO2 of 50 mmHg, PaO2 of 55 mm Hg, and SatO2 of 85%. What would be the most appropriate action at this time?
  A. Increase the FiO2 to 0.80
  B. Increase the TV to 100 ml
  C. Increase the RR to 24
  D. Increase the levels of PEEP
  E. I don’t know
26. After some days, the patient’s ventilation modality was changed to PCV with controlled pressure of 25 cmH2O, PEEP of 12, and RR of 18. The I/E ratio was 1/1. Initially, with these parameters, the TV was 380 ml on average. Two days later, the average increased to 450 ml. What is the best explanation for the change in TV in this patient?
  A. An increase in patient’s respiratory effort
  B. An increase in pulmonary compliance
  C. An increase in dead space
  D. A retention of volume leading to auto-PEEP
  E. I don’t know

## AUTHOR CONTRIBUTIONS

Tallo FS was responsible for the study conception, data curation, formal analysis, funding acquisition and study investigation. Lopes RD was responsible for the study methodology and support, and manuscript writing and review. Abib SCV supervised the study and was responsible for study support and manuscript writing and review. Baitello AL was responsible for the study investigation and support, project administration and support, manuscript drafting and writing.

## Figures and Tables

**Table 1:** Comparison of the distribution of final scores obtained within the sample of students and senior physicians.

	N	Score (Mean)	Mann-Whitney
Student	60	2.85	
Senior	60	16.16	*p*<0.0001

*p*-value: relating to the Mann-Whitney test for the comparison of the values between the 2 groups.

**Table 2:** Distribution of the medical students group by institution.

Medical schools	N	%
UNIFESP	123	22.2
UFMT	31	5.6
UFMS	29	5.2
UNINOVE	23	4.2
VOTUPORANGA	45	6.3
FAMERP	90	16.2
UNIevangélica	63	11.4
UNIFACS	11	2.0
UFGO	21	3.8
UFBA	48	8.7
UFRR	70	12.6
Total	554	100.0

UNIFESP-Universidade Federal de São Paulo, UFGO-Universidade federal de Goiás, UFBA-Universidade federal da Bahia, UFRR-Universidade federal de Rorâima, FAMERP-Faculdade de Medicina de São José do Rio Preto, UFMT-Universidade federal de Mato Grosso, UFMS-Universidade federal de Mato Grosso do Sul, UNIFACS-Universidade de Salvador.

**Table 3:** Factor Matrix.

	F1	F2	F3	F4	F5	Specificity
ARDS VENTILATION	0.957	0.008	0.081	0.098	0.118	0.05
PLATO PRESSURE IN ARDS	0.931	0.005	0.095	0.029	0.161	0.10
MECHANICAL VENTILATION COMPLACENCY	0.920	0.022	0.228	0.123	0.055	0.08
PLATO PRESSURE MEASUREMENT	0.918	0.050	0.114	0.064	0.230	0.09
PEEP	0.906	0.029	0.045	0.056	0.050	0.17
AUTO-PEEP COMPLICATIONS	0.051	0.949	0.186	0.137	0.054	0.04
AUTO-PEEP COMPLICATIONS MANAGEMENT	0.035	0.947	0.154	0.168	0.057	0.05
CPOD MECHANICAL VENTILATION	0.006	0.886	0.333	0.015	0.118	0.09
NON INVASIVE VENTILATION	0.243	0.813	0.169	0.047	0.077	0.24
AIRWAY RESISTENCE	0.145	0.302	0.832	0.064	0.174	0.16
MECHANICAL VENTILATION PRESSURES	0.263	0.346	0.754	0.127	0.035	0.23
PNEUMOTHORAX	0.314	0.188	0.690	0.294	0.129	0.29
AUTO-PEEP MEASUREMENT	0.100	0.441	0.677	0.369	0.068	0.20
CPOD VENTILATION COMPLICATIONS	0.156	0.405	0.627	0.105	0.300	0.32
VOLUME CONTROL VENTILATION	0.103	0.064	0.170	0.933	0.188	0.05
PRESSURE CONTROL VENTILATION	0.084	0.147	0.079	0.943	0.166	0.05
PRESSURE SUPPORT VENTILATION	0.146	0.126	0.117	0.769	0.340	0.25
PaO_2_	0.238	0.121	0.063	0.304	0.887	0.05
PaCO_2_	0.237	0.104	0.082	0.278	0.880	0.07
Percentage (%) of total variance explained	0.249	0.206	0.154	0.150	0.106	
Cumulative Percentage (%) of Total Variance Explained	0.249	0.455	0.609	0.759	0.865	
Alpha de Cronbach	0.909	0.906	0.715	0.830	0.906	

**Table 4:** Final score means stratified by the factors.

	Mean	Standard Deviation	Total
F1	19%	33.81	553
F2	27%	36.67	549
F3	16%	24.64	550
F4	26%	37.97	554
F5	54%	47.65	553

Average percentage for each factor (scores 0-100).
